# Carbon and Nitrogen Mineralization in Dark Grey Calcareous Floodplain Soil Is Influenced by Tillage Practices and Residue Retention

**DOI:** 10.3390/plants10081650

**Published:** 2021-08-11

**Authors:** Nazmus Salahin, Md. Khairul Alam, Sharif Ahmed, Mohammad Jahiruddin, Ahmed Gaber, Walaa F. Alsanie, Akbar Hossain, Richard W. Bell

**Affiliations:** 1 Bangladesh Agricultural Research Institute, Gazipur 1701, Bangladesh; nsalahin@bari.gov.bd (N.S.); khairul.krishi@gmail.com (M.K.A.); 2 Bangladesh Agricultural Research Council, Dhaka 1208, Bangladesh; 3 Centre for Sustainable Farming Systems, Future Food Institute, Murdoch University, Perth, WA 6150, Australia; r.bell@murdoch.edu.au; 4 International Rice Research Institute, Bangladesh Office, Dhaka 1213, Bangladesh; s.ahmed@irri.org; 5 Department of Soil Science, Bangladesh Agricultural University, Mymensingh 2202, Bangladesh; m_jahiruddin@bau.edu.bd; 6 Department of Biology, College of Science, Taif University, P.O. Box 11099, Taif 21944, Saudi Arabia; 7 Department of Clinical Laboratories Sciences, The Faculty of Applied Medical Sciences, Taif University, P.O. Box 11099, Taif 21944, Saudi Arabia; w.alsanie@tu.edu.sa; 8 Bangladesh Wheat and Maize Research Institute, Dinajpur 5200, Bangladesh

**Keywords:** carbon mineralization, carbon sequestration, mineral nitrogen, organic carbon, residues retention, total nitrogen

## Abstract

Very little is known about the changes that occur in soil organic carbon (SOC) and total nitrogen (TN) under an intensive rice-based cropping system following the change to minimal tillage and increased crop residue retention in the Gangetic Plains of South Asia. The field experiment was conducted for 3 years at Rajbari, Bangladesh to examine the impact of tillage practices and crop residue retention on carbon (C) and nitrogen (N) cycling. The experiment comprised four tillage practices—conventional tillage (CT), zero tillage (ZT), strip-tillage (ST), and bed planting (BP) in combination with two residue retention levels—increased residue (R_50%_) and low residue (R_20%_—the current practice). The TN, SOC, and mineral N (NH_4_^+^-N and NO_3_^−^-N) were measured in the soil at different crop growth stages. After 3 years, ZT, ST, and BP sequestered 12, 11, and 6% more SOC, and 18, 13, and 10% more TN, respectively than the conventional crop establishment practice at 0–5 cm soil depth. The accumulation of SOC and TN was also higher compared to the initial SOC and TN in soil. Among the tillage practices, the maximum SOC and TN sequestration were recorded with ST and with R_50%_ that might be attributed to reduced mineralization of C and N in soil particularly with increased residue retention, since decay rates of potentially mineralizable C was lower in the ST with both the residue retention practices. Increased residue retention and minimum tillage practices after nine consecutive crops has altered the C and N cycling by slowing the in-season turnover of C and N, reducing the level of nitrate-N available to plants in the growing season and increasing retained soil levels of SOC and TN.

## 1. Introduction

Changing management practices, climate variables, and input use may alter the soil biogeochemical processes [1,2,3,4]. While contributing to global food security, agriculture in the entire Gangetic region, and other paddy-growing areas where rice-anchored upland-wetland cropping systems have been practiced, has significant contributions to global greenhouse gas emissions [5,6,7]. The major terrestrial pool of C, N, and other nutrients comes from soil organic matter (SOM) while these elements are dynamically cycled through continuous changes by microbial immobilization and mineralization [8]. The potential soil productivity can be measured critically by the rate of organic C and N mineralization and the equilibrium levels maintained in different soils [1,2,9]. The biogeochemical cycles of C and N in the paddy-growing ecosystem are very active due to alternate wetting and drying of soils, resource consumptive agricultural practices, injudicious agricultural input use while striving for higher yield goals, high cropping intensity, etc. Prediction of soil C and N mineralization when crop residues are retained and build up soil organic matter in wetland soils may improve the profitability and sustainability of agriculture by allowing farmers to decrease the input of N fertilizer that optimizes crop yield. Otherwise, inefficient use of N fertilizer may cause undesirable environmental impacts, mainly through gaseous N losses by denitrification and/or ammonia volatilization [10].

Commonly, wetland rice is grown each year in a rotation with upland crops (pulses/oilseeds/vegetables/wheat/maize/potatoes, etc.,) in the Gangetic plains of South Asia. Novel resource-saving technologies are being developed for most of the crops in the rice-based cropping systems in the ecosystem [8,11,12,13]. Across the Gangetic plain, the adoption of CA practices by farmers is on the rise [11,14,15]. Growing two or three wetland rice crops continuously in an annual rotation increases SOC and TN, even where all crop residues are removed [16,17]. The increase in SOC and TN may be attributed to the short fallow period, incomplete soil drying and re-oxidation, and the return of residues of each crop of the intensive cropping systems [2]. In contrast, C and N levels appear to decline in most rice-upland crop systems such as rice-wheat [18]. Kirk and Olk [2] and Liping and Erda [19] found that under submerged conditions, both the decomposition of organic residues and the mineralization rates of residues and inherent SOM are considerably retarded in comparison with the upland conditions. Zhou et al. [20] reported C and N cycling in the paddy-upland rotation is generally harmful to soil C and N storage under the current practice of farmers [1,2]. Current crop establishment practices i.e., puddling, expedite the decomposition processes due to increased oxidation over time [21,22] which sometimes resulted in higher mineralization of soil TN. On the other hand, disturbance of soil using zero tillage (ZT) together with surface application of crop residue decreased the N mineralization rate [23]. During the upland crop period, the redox potential is increased, thereby changing the effective use of N [24]. Takahashi et al. [25] found that the degrees of increase in N uptake by all crops and increase in gross rates of N mineralization by the continuous straw application were higher than the degree of increase in total N in the soil. Most of the previous studies reported the C and N cycling either for upland crops or for a single rice crop but not after a sequence of crops grown in rice-based cropping systems.

The potentially mineralizable C (PMC) and N (PMN) pools in soil are regarded as the standard measure of the soil mineralizable C and N [26,27]. The size of these pools and associated mineralization rate constants are estimated in long-term incubation studies by fitting cumulative mineralization data in kinetic models [28,29]. Among the variety of kinetic models, the first-order model [1,2,30,31,32] and the parallel first- and zero-order kinetic model [33] are the most commonly used. The parallel first- and zero-order kinetic model [33] assumes that the soil organic matter comprises a potentially mineralizable pool of C and N (the pools that mineralize exponentially according to first-order kinetics) and a more resistant fraction that is mineralized according to zero-order kinetics [33]. For comparative purposes, most of the studies extrapolate C and N mineralization data based on an incubation study [34]. Very few studies are conducted on changes in soil C and N pools under field conditions where organic inputs vary from crop to crop and season to season [1,32]. Even fewer studies have been conducted on C and N mineralization rates in field conditions under Conservation Agriculture practices for upland and rice crops in contrast with conventional practice/traditionally puddled soils. The main objective of this study was, therefore, to determine the effect of crop establishment methods and increased crop residue retention on C and N turnover and to understand C and N dynamics in soils under the novel crop establishment technique for upland crops in a rice-upland triple-crop rotation.

## 2. Results

### 2.1. Residue Retention

In the cropping year of 2012–2013, the higher system residue retention was found in CT with 50% residue retention (6.91 t ha^−1^) followed by ST with 50% residue retention (6.66 t ha^−1^) and the lowest (5.03 t ha^−1^) was observed in BP practice (Table 1). In the following two cropping years, the highest system residue retention was recorded from the ST and BP practices with 50% residue retention (Table 1). The lowest residue retention was recorded in CT. Rice residue contributed the largest proportion of system residue inputs.

### 2.2. Crop N Content

Across tillage systems, increased (R_50%_) residue always increased crop N content for rice and lentil but not in jute, compared to low residue retention (R_20%_) (Table 2). Among the crops, the highest N recycling was recorded from the jute and the lowest from the lentil. The crop N content was not affected by the tillage practices for any crop or year.

### 2.3. Microbial Biomass Carbon (MBC)

**Figure 1:**
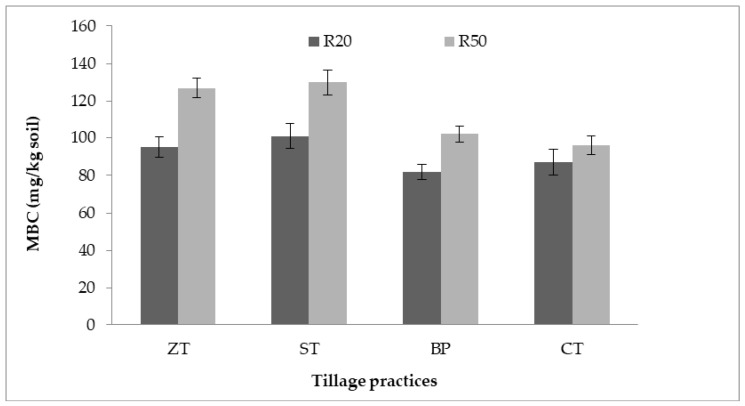
Microbial biomass carbon (MBC) in soil (0–5 cm) after 3-years under different tillage practices and residue retention level. R20 = 20% crop residue retention; R50% = 50% residue retention; ZT = zero-tillage, ST = strip-tillage, BP = bed planting, CT = conventional tillage.

The highest MBC in 0–5 cm soil depth was recorded in ST, followed by ZT and BP whereas the higher residue (R_50%_) had higher MBC values than lower residue (Figure 1).

### 2.4. Soil Organic Carbon and Total N Stock in Soil

#### 2.4.1. Soil Organic Carbon

The SOC stocks at varying depths of soil were not affected by the interaction of tillage systems and residue retention, however, their individual effects significantly changed SOC stocks (Table 3). After three years, at 0–5 cm soil depth, ZT accumulated the highest SOC (5.93 t ha^−1^), followed by ST (5.84 t ha^−1^). The SOC stock of the topsoil (0–5 cm) in ZT, ST, BP, and CT increased by 24.5%, 23.2%, 17.1%, and 11.0%, respectively, compared to the initial SOC stock (4.74 t ha^−1^). Under ST and ZT, and to a lesser extent under BP, there was a higher accumulation after 3 years of SOC at 0–5 cm soil depth, but not in the deeper soil depths (Table 3). After 3 years, increased residue retention (R_50%_) had higher SOC accumulated at all depth than the practices of low residue retention (R_20%_).

#### 2.4.2. Total N Content in Soil

After 2nd and 3rd years, soil TN content was significantly affected by the residue retention levels in all soil depth except 10–15 cm depth after the second year (Table 4). Increased residue increased the TN by 22%, 13%, and 18% at 0–5, 5–10, and 10–15 cm soils depth, respectively over initial N stock after 3 years. On the other hand, low residue retention had 9%, 8%, and 6% increased N stocks at 0–5, 5–10, and 10–15 cm soil depths, respectively, relative to the initial N stocks after 3-year (Table 4). After the 3rd year, the TN content in the soil in ZT (0.531 t ha^−1^), ST (0.509 t ha^−1^), and BP (0.496 t ha^−1^) exceeded the TN stock in CT practice (0.450 t ha^−1^). On the other hand, with increased soil depths (5–10 and 10–15 cm), the N stock did not vary among tillage practices.

### 2.5. Mineralization

#### 2.5.1. Tillage and Residues on C Mineralization

**Figure 2:**
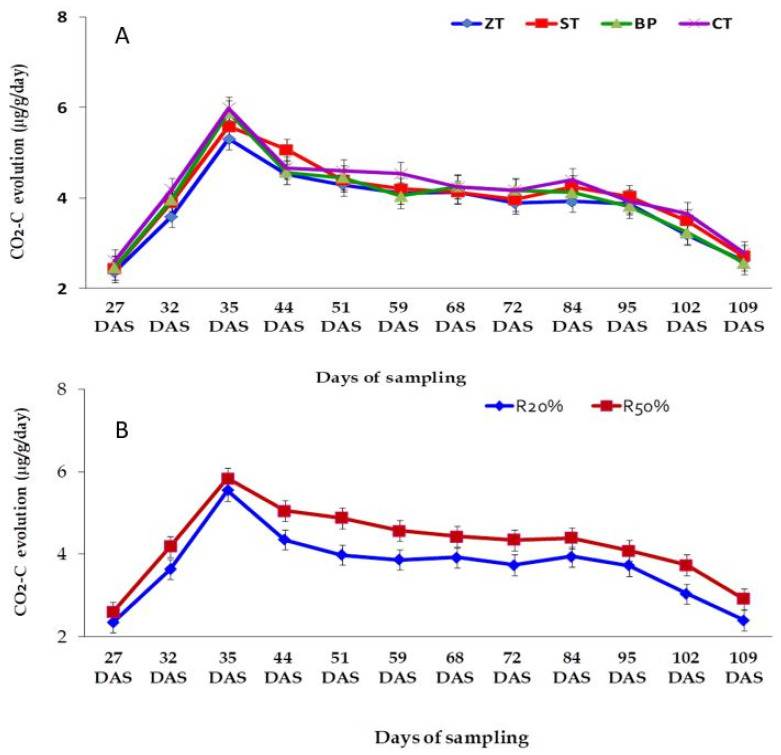
Effects of tillage practices on CO2-C evolution over time after sowing of 2nd year lentil crop. (**A**) represents CO2-C evolution under tillage practices and (**B**) represents CO2-C evolution under residue reten-tion practices. ZT, zero-tillage; ST, strip-tillage; BP, bed planting; CT, conventional tillage, R_20%_, 20% residue retention; R_50%_, 50% residue retention.

The rate of CO_2_–C evolution increased up to 35 DAS, steadily declined until 84 DAS and thereafter it sharply declined. Different tillage systems had no significant variations in CO_2_–C evolution. However, cumulative CO_2_–C emission followed the sequence as CT > BP > ST > ZT (Figure 2A).

On the other hand, the rate of CO_2_–C emission significantly increased with higher residue retention than with lower residue retention at all sampling dates (Figure 2B). There were no significant variations in the CO_2_–C emission due to the tillage and residue retention interaction.

#### 2.5.2. Kinetic Model of C Mineralization

Three parameters namely Co (easily mineralizable C pool), Kf (mineralization rate constant of the easily mineralizable C pool), and Ks (mineralization rate constant of the resistant C pool) have been estimated (Table 5). The Co pool did not vary significantly due to different tillage practices, residue retention levels, and their interaction.

There was a significant variation in Kf due to different tillage practices, residue retention levels, and their interaction (Table 5). The highest Kf value was noted in CT (0.0125%) which was significantly higher than all other tillage practices. On the other hand, a higher Kf value (0.107%) was in a lower residue retention level than that in a higher residue retention level (0.0095%). In addition, the highest Kf value was found in CT with both levels of residue retention and the lowest Kf value was noted in BP with 50% residue retention.

The mineralization rate constant of the resistant C pool (Ks) varied significantly between residue retention levels irrespective of tillage practices as well as tillage and residue interactions (Table 5). The R^2^ values were all close to 1.0 which indicated that the selected model described the mineralization process satisfactorily (Table 5).

#### 2.5.3. N Mineralization

Residue retention showed significant variations in soil NH_4_^+^-N and NO_3_^−^-N concentration regardless of tillage practices as well as tillage and residue interactions (Table 6 and Table 7). 

The increased amount of crop residue retention produced a higher amount of NH_4_^+^-N over a lower residue retention level at all sampling dates except at 102 and 109 DAS (Table 6). Unlike NH_4_^+^-N concentration, the increased amount of crop residue retention produced a lower soil NO_3_^−^-N concentration over a lower residue retention level (Table 7).

#### 2.5.4. Kinetic Model of N Mineralization

Three parameters namely, No (easily mineralizable N pool), Nf (decay rate of easily mineralizable N pool), and Ns (decay rate constant of the resistant N pool) were estimated (Table 8). The easily mineralizable N pool (No) showed no significant variations due to different tillage practices, residue retention levels, and their interaction (Table 8). However, a higher Nf value was found in lower residue retention level than with the higher residue retention level (Table 8). By contrast, a higher value of Ns was found in increased residue retention level (Table 8). The R^2^ values at all cases approach 1.0 for tillage practices, residue retention levels, and their interactions, which indicated that the selected model could describe the mineralization process satisfactorily.

## 3. Discussion

### 3.1. Effect of Tillage Practices and Residue Retention on Soil and Plant N Content and Soil C

The establishment of crops following minimal soil disturbance (ST and ZT) and increased residue retention in rice-based cropping systems increased SOC and TN accumulation over three years. While the effects of increased residue retention were evident within 2 years and to 15 cm depth after 3 years, the minimum soil disturbance effects were confined to 0–5 cm depth after 3 years. The increase of SOC and TN might be associated with the role of residues as soil cover, with decreases in disturbance of soil, increased residue return to soils; and with growing diverse crops in rotation [1]. Although increased residue retention under CA-based cropping had higher C and N accumulation after 3 years, low residue retention under CT only maintained or slightly increased SOC and TN, suggesting that increasing residue retention is necessary in this cropping system for soil C and TN accumulation. Six et al. [35] reported SOC content increased by up to 44% year^−1^ under ZT condition relative to CT in tropical and temperate countries. Alam et al. [2] reported an increase in TN in the ST system by 9 to 32% relative to CT with the increased residue retention and by 62% in ST relative to the farmers’ current practice in the Eastern Gangetic Plain on a silty clay soil after 5 years. Sapkota et al. [36] reported three times higher SOC accumulation under increased residue retention and CA-based tillage compared to conventional practices. Powlson et al. [37] reported that, in the Indo-Gangetic Plain, SOC increased at the rate of 0.16 to 0.49 t C ha^−1^ yr^−1^ with minimal soil disturbance and increased crop residue retention. In another study, minimal soil disturbance and retention of 30% of crop residues increased SOC accumulation [38].

The study confirms that the CA principles alone or in combination increase SOC and TN in soil. Some studies attributed soil TN storage to slower SOM and residue decomposition (the N in SOM, in the newly retained crop residues and in jute litterfall) because of partial contact of residues with minimally disturbed soil and with soil microorganisms [2,39,40]. The return of N in soils with residue retention also increased N input to soils. Almost half of the rice residues, litterfall, and all of the non-rice residues were retained directly on the soil under the R_50%_ practice which contributes an extra 30–50 kg ha^−1^ N return in a year in rice-based systems [2]. In a similar study, [41] reported that N accumulation can be up to 3.6 times higher with increased residue retention compared to low residue retention during the first three years. Many studies found that rapid accumulation of TN usually takes place when residues (stems, leaves, and roots) are retained at a higher rate or as standing crop stubble under minimal soil practices compared to residue removal [2,41]. Under increased residue retention, ZT and ST practices had higher N accumulation than CT and BP which in the present study was attributed to greater soil disturbance (for three crops in a year) for the raised bed preparation and accordingly incorporation of around 30–40% of the residues left on the surface. Due to the soil disturbance involved with bed re-shaping, residues retained in bed planting undergo enhanced mineralization and TN loss [2,36].

### 3.2. Effect of Tillage Practices and Residue Retention on C Mineralization

The accumulation of SOC can be attributed to the lower release of C as CO_2_ in the minimum soil disturbance treatments in the rice-based cropping systems (Figure 2A,B). Similar results on a silty clay soil in the Eastern Gangetic Plain were found following 5 years of minimal disturbance of soils and increased residue retention in rice-based intensive crop rotations [1]. Sapkota et al. [36] reported SOC increased and soil C mineralization decreased after 7 years of direct seeding in ZT plots and in permanent raised beds in the rice-maize system. The increased SOC content in these soils can be attributed to the higher potentially mineralizable C under ST/NP/CT/BP with HR. The higher PMC under those practices indicates the slow decomposition of OM in soil and the accumulation of SOC [32]. The current study also recorded higher MBC values in ST and increased residue retention which was also associated with the accumulation of SOC in the soils. Similar results of increasing SOC and MBC in soils were reported in studies that employed high residue retention and CA-based cropping [42,43]. Hence, the increased PMC and MBC under the ZT/ST/BP combined with retention of residues seem to indicate stabilization and accumulation of SOC in the intensive cropping systems.

In the current study, the quantity of CO_2_–C emitted was greater in the higher residue retention than that in lower retained plots notwithstanding the greater SOC accumulation with higher residue retention. Evidence from field studies has suggested that the rates of the CO_2_ content from decomposing plant residues added to soil are proportional to the amounts initially added [44], on moisture and temperature [45,46], on the quality of the residue/litter, e.g., lignin concentrations [47] and C: N ratio [48]. Rice straw residues have high C: N ratios compared to other crop residues in the system [49]; nevertheless, rice straw represents an important C and N source in rice crop [50]. After tillage operation, soil CO_2_–C efflux remains greater due to rise in temperature of tilled soil [51]. Compared to placing the straw on the soil surface, burying of straw in soil under CT has also been reported to accelerate the decomposition [52].

### 3.3. Effect of Tillage Practices and Residue Retention on N Mineralization

Mineralization of organic N depends on many factors such as the N requirements of the soil microbial population, the chemical composition of the decomposing crop residue, and environmental factors (e.g., temperature changes) [45]. ZT practice lowers mineralization and nitrification rates and increases immobilization of N [53]. In the present study, different tillage practices showed no significant variations in NH_4_^+^-N content in field conditions. The findings were consistent with the previous reports of Malhi and Lemke [54], who observed that tillage did not affect soil NH_4_^+^-N. On the other hand, the findings of Dong et al. [55] are contradictory to our results as they noted that the soil NH4^+^-N content was likely greater for ZT because of lower soil ammonium consumption by microorganisms under such cultivation than CT. Similarly, López-Bellido et al. [56] stated that the ZT produced greater soil NH_4_^+^-N levels than CT because of lower microbial consumption by microbes and higher physical protection of organic material within macro-aggregates formed in ZT compared with CT.

The NH_4_^+^-N content was significantly higher with increased crop residue retention levels but increased residue retention had the opposite effect on soil NO_3_^−^-N. The NH_4_^+^-N results can be attributed to the increased amount of previous crop residue retention that not only supplied more N to the soil, but also preserved more soil moisture and lowered soil temperature but it is not clear why soil NO_3_-N was higher with lower residue retention.

In the present study, different tillage practices had no significant differences in soil NO_3_^−^-N content in the field. Many researchers reported that intensive tillage enhanced soil aeration and ultimately led to the formation of NO_3_^−^-N [57,58,59]. The greater NO_3_^−^-N content under CT compared with ZT was observed by López-Bellido et al. [56]. Intensive soil tillage accelerates N mineralization of crop residues and soil organic N [60] and increases the accumulation of NO_3_^−^-N in the soil profile [61]. Malhi and Lemke [54] reported that tillage greatly affected soil NO_3_^−^-N at 0–5 cm depth, where ZT had significantly lower NO_3_^−^-N relative to CT. Less NO_3_^−^-N N in ZT soil was possible because with less soil disturbance the organic N mineralization was significantly reduced and thus the concentration of NO_3_^−^-N decreased [62]. The minimum soil disturbance treatments (ST and ZT) may need to continue for longer than 3 years on the sandy loam soils to lower soil NO_3_^−^-N as found in many prior studies.

### 3.4. Effect of Tillage Practices and Residue Retention on C and N Cycling

The disturbance of soil increased the decay rate of easily or potentially mineralizable C over the crop-growing period. On the contrary, ST had the highest PMC, probably due to the lower decay rate and higher SOM content in the soil [32,52]. BP and CT had a higher decay rate than ZT or ST and hence lower PMC, probably because these types of crop establishment had not accumulated as much SOC as ST and ZT.

By contrast with C, tillage practices had no significant effect on the potentially mineralizable N (PMN) pool, the decay rate of PMN, and the decay rate of the relatively resistant pool. Since CT caused the physical destruction of crop residues, increased the soil-residue contact, promoted higher aeration and higher soil temperature, over time there is likely to be increased soil N mineralization compared to minimum soil disturbance [63]. After five years of tillage and residue retention practices under mustard-irrigated rice-monsoon rice and wheat-jute-monsoon rice cropping systems, Alam et al. [2] found strip planting alone and in combination with increased residue retention have significantly higher PMN pool (depending on crop and season, 15–29% higher) than conventional practice which they attributed to lower decay rate of PMN due to minimal soil disturbance, increased biomass production, less soil temperature and less residue contact with soil.

Decay rates of PMC under ST and ZT under increased residue retention were lower than the decay rate of PMC in conventional tillage and increased residue retention (Table 5). The increased rate of residue retained in the soil under minimal soil disturbance practice led to higher SOM content over the three years and a lower decay rate of PMC. Accordingly, increased residue retention gave the highest PMC after running into the zero-order and first rate exponential pool [32,64]. Alam et al. [1] reported that the SP soils recorded higher Co values (relative to CT practice) under all crops in rice-upland triple cropping systems after 5 years, probably because of the higher C contents, and lower PMC decay rate.

Alam et al. [2] recorded that increased residue retention had higher decay rate but higher PMN value under all tillage practices which they related to increased rate of residue retention. The present study also showed that retention of residue at an increased rate had higher PMN. Increased rate of high residue retained in the soil had higher TN content and a lower decay rate of PMN than low residue retention. Accordingly, increased residue retention gave the numerically highest PMN according to the model (zero-order and first rate exponential pool) used [32,64].

## 4. Materials and Methods

### 4.1. Location and Description of the Experimental Site

A cropping systems-based experiment was conducted in a farmer’s field (23°39″45′ N, 89°29″39′ E) at Baliakandi Upazila, Rajbari district, Bangladesh from July 2012 to July 2015, as described by [13]. The experiment site is under Agro-Ecological Zone 12 (Low Ganges River Floodplain) with a well-drained Calcareous Dark Grey Floodplain Soil (FAO: Chromic-Calcaric Gleysols). The soil texture class was sandy loam.

Among the major cropping systems, rice-lentil-jute was selected in the current study [35]. Rice growing period was from July to November, the lentil growing period was from November to March and the jute growing period stretched from March to July. The trial was initiated with rice (transplanted aman rice) in July 2012. Before starting the first crop, the initial soil properties of the experimental field were analyzed at 0–5, 5–10, and 10–15 cm soil depths. Initial soil properties can be found in [13]. In the initial soil the SOC stocks were 4.76, 4.36, and 3.95 t ha^−1^, total N were 0.430, 0.398, 0.364 t ha^−1^, and microbial biomass carbon (MBC) were 122, 115, and 110 mg kg^−1^, respectively, for 0–5, 5–10, and 10–15 cm soil depth. The SOC was determined using the wet oxidation method [65], total N was determined using the Kjeldahl method [66], MBC was determined using the chloroform fumigation–incubation method [67].

### 4.2. Design and Treatments

The study included four tillage practices—for the upland crop (i) zero-tillage (ZT), (ii) strip-tillage (ST), (iii) bed planting (BP), and (iv) conventional tillage (CT), and two levels of crop residue retention—low residue (R_20%_) and high residue (R_50%_). However, for the rice crop, the practices were zero tillage non-puddling (NPZT), non-puddling followed by ST (NPST), non-puddling followed by BP (NPBP), and conventional puddling (CT). The experiment was laid out in a split-plot design and the main plot treatment was tillage practices and the sub-plot treatment was residue retention level with four replications. Each sub-plot size was 9.0 m × 6.0 m. The beds were prepared for the first crop and they were reformed for every subsequent crop over the experimental period. Residues of rice and wheat were retained by 20% and 50% based on height, during the harvesting period, while for the lentil the retention levels were according to weight of the stover. For jute, all of the fallen leaves were dropped on the soil during its growing period regardless of residue treatment. The details of residue retention can be found at Salahin et al. [13].

### 4.3. Crop Sequence and Variety

The crop sequence was *aman* rice (*Oryza sativa* L.)—lentil (*Lens culinaris* Medikus)—jute (*Corchorus olitorius*) for the first two years and in the 3rd year wheat (*Triticum aestivum* L.) replaced lentil. The varieties of the crops were: for rice cv. Binadhan-7, for lentil cv. BARI Mosur-3, for jute cv. Nabin (JRO-524) and for wheat cv. BARI Gom-26. Those varieties were selected based on the climatic suitability and popularity among the farmers.

### 4.4. Crop Management

Land preparation for ZT, ST, BP, and CT are reported in detail by [13]. The crop management and rates of chemical fertilizers for component crops along with their application methods in the cropping sequence are detailed in Salahin et al. [13]. Weeds, insect and disease control was done as and when required following the standard safety guidelines. The details of pest management can be found in [13].

### 4.5. Data Collection

#### 4.5.1. Plant N Analysis

Rice, lentil, and jute crops (first two years) were destructively sampled to determine the biomass weight and N concentration in shoots. For the rice, four pre-marked hills from each plot were harvested at physiological maturity. After air drying, the samples were oven-dried at 70 °C for 48 h then weights converted to dry matter plot^−1^ based on the total hills plot^−1^. For jute and lentil, 10 pre-marked plants were harvested just immediately before harvesting and then the samples were air-dried before oven-drying at 70 °C for 48 h and then converted to dry matter m^−2^. The biomass of all crops was then converted to t ha^−1^. Nitrogen concentrations in the plant samples were determined by the Kjeldahl method [66]. Plant uptake of N was calculated by multiplying the N concentration in shoots by the dry biomass to give N content. The N uptake by each crop was converted to the percentage of total N available (see below for details of this parameter) at the respective stage.

#### 4.5.2. Microbial Respiration

Microbial respiration was assessed by measuring CO_2_ evolution from the soil in the lentil field during the rabi season by trapping emitted CO_2_ in NaOH [41]. The trapped CO_2_ was measured by adding 15 mL of 10% *w*/*v* BaCl_2_ to the NaOH to precipitate BaCO_3_. The remaining NaOH was then back titrated against 1M HCI to the phenolphthalein end point to neutralize NaOH. Finally, more HCI was added to the solution to dissolve BaCO_3_. The CO_2_ amount was determined by using the following (Equation (1)):

Evolved CO_2_ (mg day^−1^) = {(T2 − T1) × M × 22)}/t (1)

where, T_1_ = HCI amount used to neutralize NaOH, T_2_ = T_1_ + HCl amount used to dissolve precipitated BaCO_3_, M = molarity of HCl, 22 = 22 mg CO_2_/l mL 1M HCl, and t = time in days.

#### 4.5.3. Nitrogen Availability Measurements

Both NH_4_^+^ and NO_3_^−^ nitrogen were extracted from the soils with 1 M KCl to determine the extractable mineral N in the soil sample [68]. Based on the bulk density of the soil, the NH_4_^+^ and NO_3_^−^ nitrogen were then converted to kg ha^−1^ [69]. The total N uptake values for crops at each soil sampling date were added to the measured amount of extractable N (NH_4_^+^ + NO_3_^−^) recorded in soils to determine the amount of available N.

#### 4.5.4. Modeling of SOC and N Mineralization Data with the Parallel First and Zero-Order Kinetic Model

The related data were assessed using the parallel first and zero-order kinetic model [31,33], assuming that SOC comprises an easily mineralizable C pool that mineralized exponentially according to the first-order kinetics, while the resistant C fraction mineralizes following zero-order kinetics [33]. The integrated equation is therefore written as follows (Equation (2)), assuming that the resistant C fraction is not diminished significantly during the study period.

Ct = Co1 − exp – kf × t + Ks × t(2)

where, Ct is the cumulative amount of C mineralized at time t, Co is the amount of easily mineralizable C pool expressed in mg C g^−1^, Kf is the mineralization rate constant of the easily mineralizable C pool, Ks is the mineralization decay rate of resistant C pool, and t is the time.

The N mineralization data were also assessed using a comparable kinetic model (Equation (3)), assuming that the resistant N fraction is not diminished significantly during the study:

Nt = No1 − exp – Nf × t + Ns × t(3)

where, Nt indicates the net N mineralized at a definite time (t), No is the easily mineralizable N pool (mg N g^−1^), Nf is the mineralization rate constant of the No, Ns is the decay rate of resistant N pool, and t is the time.

### 4.6. Climate and Weather

The climate of the area is subtropical. From November to March is the dry period when almost no rainfall occurs and the temperature remains low especially during December and January. April to September is the hot and wet period and the maximum rain falls during July and August. The total rainfall during the trial period was 1751, 1510, 1850 mm in the cropping years of 2012–2013, 2013–2014, and 2014–2015, respectively. The daily minimum temperature was lower in the cropping year 2012–2013 than the other two years. Daily temperatures, rainfall, and sunshine hour data were collected from the nearest weather station (Faridpur; 35 km away) to the trial field and shown in [13].

### 4.7. Statistical Analysis

All data related to crop and soil were statistically analyzed using a split-plot model. The treatment effects on different parameters were tested by analysis of variance (ANOVA), and treatment means comparisons among the treatments were made using the least significant difference (LSD) tests at a 5% level of probability (*p* < 0.05). Statistical procedures were carried out with the software program Statistix (Statistix Inc., Tallahassee, FL, USA) [70]. The C and N mineralization data and kinetic parameters data were fitted by parallel first and zero-order kinetic functions using the SPSS Inc. software (SPSS Inc., Chicago, IL, USA).

## 5. Conclusions

Both minimum soil disturbance practices and increased crop residue retention played a significant role in sequestering SOC and TN in the sandy loam soil after 3 years. After nine consecutive crops, increased residue retention with minimum soil disturbance practices (ZT, ST) and with BP has altered the C and N cycling. Among the tillage practices, the maximum SOC and TN sequestration were recorded in ST at 0–5 cm depth that might be attributed to reduced mineralization of C and N in soil under this practice, since decay rates of potentially mineralizable C were also lower in the ST with both the residue retention practices. Increased residue retention with minimum tillage practices after nine consecutive crops slowed the in-season turnover of C and N, reducing the level of nitrate-N available to plants in the growing season and increasing retained soil levels of SOC and TN.

## Figures and Tables

**Table 1:** Amount of residue retained (t ha^−1^) from different crops (except jute leaf litter) and total systems residue retention in the cropping years, 2012–2013, 2013–2014, and 2014–2015.

	Cropping Year 2012–2013								Cropping Year 2013–2014								Cropping Year 2014–2015							
Tillage System	Rice		Lentil		Jute		System		Rice		Lentil		Jute		System		Rice		Wheat		Jute		System	
Residue	R_20%_	R_50%_	R_20%_	R_50%_	R_20%_	R_50%_	R_20%_	R_50%_	R_20%_	R_50%_	R_20%_	R_50%_	R_20%_	R_50%_	R_20%_	R_50%_	R_20%_	R_50%_	R_20%_	R_50%_	R_20%_	R_50%_	R_20%_	R_50%_
ZT	1.73	2.88	0.22	0.59	2.55	2.25	4.5	5.72	2.49	4.27	0.22	0.54	1.17	1.25	3.88	6.06	2.3	4.3	2.03	3.95	1.45	1.57	5.78	9.82
ST	1.90	3.58	0.26	0.59	2.33	2.43	4.49	6.66	2.50	4.47	0.24	0.56	1.42	1.26	4.16	6.29	2.4	4.6	2.08	4.08	1.55	1.68	6.03	10.36
BP	1.45	3.20	0.23	0.47	1.46	1.36	3.14	5.03	3.34	4.54	0.29	0.69	1.13	0.94	4.76	6.17	2.7	4.8	2.40	4.23	1.11	1.39	6.21	10.42
CT	2.38	3.93	0.27	0.60	2.33	2.38	4.98	6.91	2.40	3.95	0.20	0.52	1.33	1.44	3.93	5.91	2.3	4.1	2.10	3.73	1.27	1.25	5.67	9.08

ZT, zero-tillage; ST, strip-tillage; BP, bed planting; CT, conventional tillage; R_20%,_ low residue retention; R_50%_, high residue retention.

**Table 2:** Effect of tillage practices and residue retention on nitrogen content (kg ha^−1^) in crop residues retained in the rice-lentil jute cropping system.

Tillage Practices (TP)	Residue Retention (RR)	Rice			Lentil		Jute			Wheat
		1st Year	2nd Year	3rd Year	1st Year	2nd Year	1st Year	2nd Year	3rd Year	3rd Year
ZT	R_20%_	10.0	9.2	9.20	2.60	2.80	35.7	16.4	20.3	9.10
	R_50%_	18.1	18.0	15.3	6.00	5.34	32.6	18.1	22.8	17.8
ST	R_20%_	10.3	9.84	9.43	3.10	3.21	32.6	19.9	21.7	9.40
	R_50%_	18.9	19.3	15.4	7.14	6.58	35.2	18.3	24.4	18.4
BP	R_20%_	13.2	10.8	8.50	2.71	2.86	20.4	15.8	15.5	10.8
	R_50%_	18.5	19.7	15.1	5.64	6.04	19.7	13.6	20.2	19.0
CT	R_20%_	9.60	9.20	10.0	3.21	3.44	32.6	18.6	17.8	9.50
	R_50%_	15.6	16.0	13.3	7.20	7.15	34.5	26.7	18.1	16.8
LSD_0.05_-TP		NS	NS	NS	NS	NS	NS	NS	NS	NS
LSD_0.05_-RR		5.10	4.82	4.40	2.11	1.72	2.12	3.65	3.70	4.8

ZT, zero-tillage; ST, strip-tillage; BP, bed planting; CT, conventional tillage; R_20%,_ 20% residue retention; R_50%_, 50% residue retention; LSD_0.05_, least significant difference at 5% level of probability; NS, not significant.

**Table 3:** Soil organic carbon (SOC) stocks (t ha^−1^) as influenced by tillage practices and crop residues retention levels after 1st, 2nd, and 3rd year at different depths of soil.

Treatment	After 1st Year			After 2nd Year			After 3rd Year		
	0–5 cm	5–10 cm	10–15 cm	0–5 cm	5–10 cm	10–15 cm	0–5 cm	5–10 cm	10–15 cm
Tillage system									
ZT	5.09	4.51	4.10	5.61	4.80	4.16	5.90	5.03	4.34
ST	5.32	4.62	4.22	5.49	4.74	4.45	5.84	4.97	4.68
BP	4.86	4.51	4.10	5.26	4.68	4.28	5.55	4.74	4.34
CT	4.57	4.57	4.22	5.03	4.51	4.34	5.26	4.80	4.62
LSD_0.05_	NS	NS	NS	NS	NS	NS	0.26 **	NS	NS
CV (%)	11.0	4.5	4.1	6.9	8.5	10.0	5.0	9.0	11.5
Residue retention									
R_20%_	4.91	4.45	4.10	5.03	4.51	4.16	5.32	4.74	4.22
R_50%_	5.03	4.62	4.28	5.61	4.91	4.39	5.90	5.03	4.80
LSD_0.05_	NS	NS	NS	0.29 *	0.35 *	NS	0.23 *	0.17 **	0.12 **
CV (%)	4.9	6.3	5.4	7.4	9.2	7.2	5.7	4.6	2.8
The initial status of SOC was 4.74, 4.39, and 3.93 t ha^−1^ at 0–5, 5–10, and 10–15 cm soil depth, respectively.									

ZT, zero-tillage; ST, strip-tillage; BP, bed planting; CT, conventional tillage; R_20%_, 20% residue retention; R_50%_, 50% residue retention; LSD, least significant difference; NS, not significant; CV, coefficient of variation; * = *p* < 0.05, ** = *p* < 0.01.

**Table 4:** Total N content (t ha^−1^) of soil as influenced by tillage practices and residue levels after 1st, 2nd, and 3rd years of cropping.

Treatment	After 1st Year			After 2nd Year			After 3rd Year		
	0–5 cm	5–10 cm	10–15 cm	0–5 cm	5–10 cm	10–15 cm	0–5 cm	5–10 cm	10–15 cm
Tillage system									
ZT	0.486	0.424	0.380	0.500	0.431	0.383	0.531	0.452	0.393
ST	0.478	0.419	0.393	0.496	0.427	0.409	0.509	0.445	0.429
BP	0.465	0.412	0.382	0.472	0.423	0.391	0.496	0.432	0.400
CT	0.448	0.407	0.380	0.451	0.409	0.387	0.450	0.427	0.427
LSD_0.05_	NS	NS	NS	NS	NS	NS	0.054 *	NS	NS
CV (%)	4.9	7.3	7.8	8.8	6.4	7.1	7.8	4.2	4.1
Residue retention									
R_20%_	0.460	0.411	0.380	0.457	0.411	0.389	0.468	0.430	0.385
R_50%_	0.478	0.420	0.388	0.504	0.435	0.396	0.525	0.448	0.427
LSD_0.05_	NS	NS	NS	0.035 **	0.020 *	NS	0.022 **	0.009 **	0.014 **
CV (%)	5.0	7.2	8.2	9.0	6.4	6.7	8.0	4.6	4.1
The initial status of TN was 0.430, 0.398 and 0.364 t ha^−1^ at 0–5, 5–10 and 10–15 cm soil depth, respectively									

ZT, zero-tillage; ST, strip-tillage; BP, bed planting; CT, conventional tillage; R_20%_, 20% residue retention; R_50%_, 50% residue retention; LSD, least significant difference; NS, not significant; CV, coefficient of variation; * = *p* < 0.05, ** = *p* < 0.01.

**Table 5:** Estimated parameters of a fitted parallel first and zero-order kinetic model for predicting C mineralization.

Treatments	Co (mg C g^−1^ C)	Kf	Ks	R^2^
Tillage practices				
ZT	364	0.0088 b	7.05	0.998
ST	480	0.0093 b	6.10	0.998
BP	356	0.0097 b	6.84	0.998
CT	350	0.0125 a	7.25	0.999
LSD_0.05_	NS	0.001 **	NS	-
Residue retention levels				
R_20%_	358	0.0107 a	6.25 b	0.998
R_50%_	417	0.0095 b	7.37 a	0.998
LSD_0.05_	NS	0.001 *	0.62 **	-
CV (%)	28.0	8.8	13.9	-
Tillage practices × residue retention levels				
ZT × R_20%_	283	0.0087 bc	6.67	0.998
ZT × R_50%_	445	0.0090 bc	7.43	0.998
ST × R_20%_	513	0.0097 b	5.18	0.998
ST × R_50%_	447	0.0090 bc	7.01	0.998
BP × R_20%_	300	0.0117 a	6.08	0.998
BP × R_50%_	412	0.0077 c	7.59	0.999
CT × R_20%_	334	0.0127 a	7.05	0.999
CT × R_50%_	365.6	0.0123 a	7.45	0.999
LSD_0.05_	NS	0.002 *	NS	-
CV (%)	18.2	11.6	9.7	-

ZT, zero-tillage; ST, strip-tillage; BP, bed planting; CT, conventional tillage; R_20%_, 20% residue retention; R_50%_, 50% residue retention; LSD, least significant difference; CV, coefficient of variation; * = *p* < 0.05, ** = *p* < 0.01; different letters in the same column indicates significant difference; Co, amount of easily mineralizable C pool; Kf, mineralization rate constant of the easily mineralizable C pool; Ks, mineralization decay rate of resistant C pool; R^2^, correlation of determination; ”-”, not any statistical analysis. Lower-case letters in the same column indicates significantly different at 5% probability level.

**Table 6:** Effects of tillage and residues on soil NH_4_^+^-N concentration (mg kg^−^^1^) at 0–15 cm soil depth.

Treatments	Days after Sowing											
	27	32	35	44	51	59	68	72	84	95	102	109
Tillage practices												
ZT	9.7	11.4	12.3	12.7	13.8	13.2	12.3	11.4	11.1	10.7	10.3	10.2
ST	10.0	11.7	12.6	13.3	14.2	13.7	12.7	11.9	11.3	11.0	10.6	10.3
BP	10.2	11.9	12.9	13.5	14.5	13.9	13.0	11.8	11.5	11.2	10.6	10.3
CT	9.6	11.2	12.0	12.7	13.5	12.9	12.1	11.7	11.1	10.8	10.4	10.1
LSD_0.05_	NS	NS	NS	NS	NS	NS	NS	NS	NS	NS	NS	NS
CV (%)	3.8	3.3	3.7	5.9	6.1	5.7	5.9	4.9	8.7	6.3	3.9	5.5
Residue retention levels												
R_20%_	9.5	11.2	12.1	12.7	13.6	13.0	12.2	11.2	10.8	10.7	10.4	10.1
R_50%_	10.2	11.9	12.9	13.4	14.3	13.8	12.9	12.2	11.7	11.2	10.5	10.3
LSD_0.05_	0.23 **	0.35 **	0.33 **	0.62 *	0.70 *	0.70 *	0.61 *	0.72 *	0.59 **	0.39 *	NS	NS
CV (%)	2.5	3.3	2.8	5.1	3.3	5.6	5.2	6.5	5.6	3.8	3.0	2.7

ZT, zero-tillage; ST, strip-tillage; BP, bed planting; CT, conventional tillage; R_20%_, 20% residue retention; R_50%_, 50% residue retention; LSD, least significant difference; NS, not significant; CV, coefficient of variation; * = *p* < 0.05, ** = *p* < 0.01.

**Table 7:** Effects of tillage and residues on soil NO_3_^−^-N concentration (mg kg^−1^) at 0–15 cm soil depth.

Treatments	Days after Sowing											
	27	32	35	44	51	59	68	72	84	95	102	109
Tillage practices												
ZT	11.0	12.9	14.9	15.9	17.3	16.6	16.4	16.0	15.9	15.7	14.8	13.8
ST	11.5	12.4	14.7	16.0	17.6	17.0	16.9	16.1	16.0	15.9	15.3	14.1
BP	11.3	12.8	15.1	16.5	17.9	17.6	17.4	16.6	16.4	16.0	15.4	14.1
CT	12.2	13.3	15.4	16.8	18.1	17.3	17.2	16.9	16.5	16.4	15.5	14.4
LSD_0.05_	NS	NS	NS	NS	NS	NS	NS	NS	NS	NS	NS	NS
CV (%)	7.2	11.4	8.5	9.2	10.4	9.2	12.4	11.2	9.7	14.0	17.8	11.8
Residue retention levels												
R_20%_	11.9	13.6	15.7	16.9	18.2	17.7	17.4	16.9	16.6	16.2	15.6	13.9
R_50%_	11.1	12.1	14.3	15.7	17.3	16.6	16.5	15.9	15.8	15.7	14.9	14.4
LSD_0.05_	0.65 *	0.77 **	0.64 **	0.45 **	0.38 **	0.39 **	0.56 **	0.58 *	0.61 *	NS	NS	NS
CV (%)	5.2	6.4	4.6	3.0	2.3	2.4	3.5	3.8	4.0	10.8	11.1	7.7

ZT, zero-tillage; ST, strip-tillage; BP, bed planting; CT, conventional tillage; R20%, 20% residue retention; R50%, 50% residue retention; LSD, least significant difference; NS, not significant; CV, coefficient of variation; * = *p* < 0.05, ** = *p* < 0.01.

**Table 8:** Estimated parameters of a fitted parallel first and zero-order kinetic model for predicting N mineralization.

Treatments	No (mg N g^−1^ N)	Nf (%)	Ns (%)	R^2^
Tillage practices				
ZT	149	0.091	7.28	0.996
ST	158	0.098	7.12	0.996
BP	148	0.108	7.21	0.996
CT	146	0.116	7.04	0.996
LSD_0.05_	NS	NS	NS	-
CV (%)	16.2	20.4	8.8	-
Residue retention levels				
R_20%_	142	0.114	6.95	0.996
R_50%_	158	0.093	7.38	0.996
LSD_0.05_	NS	0.025 *	0.41 *	-
CV (%)	16.4	15.9	6.1	-

ZT, zero-tillage; ST, strip-tillage; BP, bed planting; CT, conventional tillage; R_20%_, 20% residue retention; R_50%_, 50% residue retention; LSD, least significant difference; CV, coefficient of variation; * = *p* < 0.05, ** = *p* < 0.01; Co, amount of easily mineralizable C pool; Kf, mineralization rate constant of the easily mineralizable C pool; Ks, mineralization decay rate of resistant C pool; R^2^, correlation of determination; ”-”, not any statistical analysis.

## Data Availability

Data are not publicly available, though the data may be made available on request from the corresponding author.
